# The Influence of Aspiration Pressure, Follicle Flushing Method and Needle Rotation During Single-Operator OPU Technique on Oocyte Recovery and Embryo Production in the Mare

**DOI:** 10.3390/ani15060832

**Published:** 2025-03-14

**Authors:** Juan Cuervo-Arango, Laura Sala-Ayala, Adrián Márquez-Moya, Rebeca Martínez-Boví

**Affiliations:** Equine Fertility Group, Faculty of Veterinary Medicine, Universidad Cardenal Herrera-CEU, CEU Universities, 46115 Alfara del Patriarca, Valencia, Spain; laura.salaayala@uchceu.es (L.S.-A.); adrian.marquezmoya@uchceu.es (A.M.-M.)

**Keywords:** horse, assisted reproductive technique, oocyte recovery, transvaginal follicle aspiration, in vitro embryo production

## Abstract

The objective of this study was to determine the effect of two aspiration pressures (75 vs. 150 mmHg), the follicle flushing method (injection pump controlled by a foot pedal vs. a plastic syringe) and the twisting of the OPU needle on oocyte recovery and in vitro embryo production. OPU data from a total of 104 warmblood sport mares belonging to a commercial OPU-ICSI program were collected as part of a prospective study split into three experiments. Each mare was used only once for OPU. In Experiment 1, the mares’ follicles were aspirated using either a high aspiration pressure (flow rate of 1.33 mL/s; *n* = 18) or low aspiration pressure (0.75 mL/s; *n* = 18); in Experiment 2, follicles were flushed using either a manual method (plastic syringe, *n* = 18) or an automatic method (injection pump controlled by a foot pedal, *n* = 18); and in Experiment 3, the follicles were aspirated by scraping the follicle wall with needle rotation (needle twisting, *n* = 16) or without needle rotation (control, *n* = 16). In all the experiments, the same OPU operator and technician searching oocytes were used, and the allocation of each mare to the different treatment groups was randomized. The overall mean oocyte recovery rate of the study was 54.2 ± 17.1%, and the mean number of embryos per OPU-ICSI session was 1.9 ± 1.6. The oocyte recovery rate was not influenced by any of the parameters investigated (*p* > 0.05). However, high aspiration pressure (150 mmHg) tended to yield oocytes with lower maturation (51.6%; *p* = 0.09) and blastocyst rates (20.6%; *p* = 0.08) following IVM and ICSI, respectively, compared with the low aspiration group (64.4% MII rate and 31.4% blastocyst rate). In conclusion, increasing aspiration pressure does not increase oocyte recovery. Furthermore, when a single operator performs the OPU (holding the ovary and handling the needle simultaneously), needle rotation to scrape the follicle wall does not improve oocyte recovery.

## Simple Summary

The in vitro production of embryos by ovum pick-up (OPU) and intracytoplasmic sperm injection (ICSI) is the most efficient assisted reproductive technique in the horse, as it provides the largest number of embryos produced per attempt. Apart from individual mare and stallion factors and ICSI laboratory experience, the number of recovered oocytes per OPU session is the most relevant factor that influences the success of the technique and the number of in vitro-produced embryos. Therefore, it is important to investigate how different variables of the OPU technique affect oocyte recovery and the quality of those oocytes to produce embryos. This study investigated the influence of vacuum pressure, follicle flushing methods, and the twisting of the OPU needle performed by a single operator on the oocyte recovery rate and embryo production in an OPU-ICSI commercial program in mares. Increasing the aspiration pressure did not improve oocyte recovery but tended to decrease oocyte developmental competence. The follicle flushing method (plastic syringe vs. injection pump) did not affect either oocyte recovery or the number of embryos. Surprisingly, the twisting of the OPU needle to aid scraping of the follicle wall did not influence oocyte recovery during the single-operator OPU technique, which possibly indicates that the scraping technique attempted by a single-operator in this study was not efficient in dislodging oocytes from follicles.

## 1. Introduction

Transvaginal follicle aspiration or ovum pick-up (OPU) is an assisted reproductive technique used in horses to obtain immature oocytes (germinal vesicle stage) for in vitro embryo production [1] by either intracytoplasmic sperm injection (ICSI) [2] or in vitro fertilization (IVF) [3]. In the last 10 years, the OPU procedure has become increasingly popular owing to a high demand from sport horse breeders to obtain in vitro-produced embryos from mares and stallions with valuable genetics [4]. The upsurge in OPU has been paralleled by an increase in the efficiency of the ICSI technique [5] and transfer of cryopreserved in vitro-produced embryos [6]. Currently, the average embryo yield per OPU-ICSI session is reported to be 2.12 [7].

One of the main factors that influences the number of IVP embryos per OPU-ICSI session is the number of aspirated follicles and recovered oocytes per mare [6]. The current OPU technique has evolved substantially from the more invasive techniques previously used, which involved the aspiration of pre-ovulatory follicles via colpotomy [8] or laparotomy [9]. Nowadays, all visible antral follicles are aspirated transvaginally while the ovary is held by the operator’s hand through the rectum, fixating it against the vaginal wall [1]. Follicle flushing is a relevant process during OPU in mares to increase oocyte recovery owing to the stronger attachment of the oocyte cumulus complex to the granulosa layer. On the contrary, follicle flushing in cattle OPU is not performed routinely, since the oocyte is not as attached to the granulosa layer as in a mare [10]. In mares, follicle flushing is usually performed by manual injection of heparinized media using a rubber-free plastic syringe (0.5 to 5 mL per follicle, depending on follicle size). More recently, a purpose-made combined infusion and aspiration (vacuum) pump for equine OPU has become commercially available (Minitube aspiration and flushing pump for equine OPU, 230 V, Tiefenbach, Germany), allowing for flushing and aspiration of follicles under the control of a foot-pedal. It was recently shown, using slaughterhouse material (postmortem ovaries), that flushing a follicle 10 times was associated with the highest oocyte recovery compared to fewer times [11]. However, it is unknown whether the flushing method (manual flushing using a plastic syringe or an injection pump) would influence oocyte recovery.

The OPU technique can also differ with respect to the number of operators needed to perform the procedure. One option is to have two operators, where one operator holds the ovary and the probe, while the second operates the needle to puncture follicles. A third operator flushes follicles using a syringe unless an infusion pump is used; then, this third operator is not required. The other option is to have a single operator who holds the ovary and probe and operates the needle at the same time; a second operator then uses a syringe or an infusion pump, eliminating the need for additional operators (solo-OPU technique). It is logical to think that one single operator cannot twist the needle as efficiently as a second operator, because the hand needs to be used to hold the probe and the needle simultaneously. Twisting the needle to scrape the follicle wall was associated with an increase in oocyte recovery of 15 to 50 percentual points in a research model using postmortem ovaries from mares [11] and cattle [12], respectively. However, this has not been investigated in live mares.

Lastly, the aspiration pressure used to collect oocytes has been shown to influence oocyte recovery in cattle: the higher the pressure, the higher the oocyte recovery [12,13,14], but the quality of the oocytes can be affected by higher vacuum pressures, which in turn can result in lower blastocyst production [13].

The objectives of this study were (1) to determine the effect of aspiration pressure on oocyte recovery and in vitro embryo production, (2) to investigate the influence of the follicle flushing method (manual vs. injection pump) on oocyte recovery, and (3) to determine the effect of needle twisting to aid follicle wall scraping in OPUs performed by a single operator on oocyte recovery. It was hypothesized that higher vacuum pressures would increase oocyte recovery but would not increase the number of embryos produced per OPU-ICSI session. Furthermore, the twisting of the needle would increase oocyte recovery owing to the positive effect of scraping on oocyte dislodgment. Lastly, the flushing method used to inject media into the follicle would not influence oocyte recovery.

## 2. Materials and Methods

### 2.1. Animals

In total, 104 privately owned warmblood showjumping mares were used in the study between 2022 and 2024, during all seasons except summer (June to September), when no OPU was performed. The mean age of the mares was 13.5 ± 5.1 years (3 to 26 years). The mares were resident at a veterinary clinic located in Spain or were brought into the clinic as external patients for the OPU procedure. All animals were part of a commercial OPU-ICSI program for in vitro production of embryos, and the owners provided informed consent for inclusion of the data generated from the program for research purposes. Each mare was aspirated only once (one-time procedure); therefore, the experiment included 104 different mares and 104 OPU procedures.

### 2.2. OPU Technique

The mares were not selected by follicle numbers, and therefore, they were not scanned prior to the OPU session to confirm antral follicle count; thus, the reproductive stage (anestrus or cyclic) or the phase of the estrous cycle (diestrus or estrus) at the time of OPU was unknown. The mares arrived at the clinic the day prior to or on the same day of the OPU session. Initial sedation was provided with 6 mg butorphanol tartrate i.v. (Butomidor, 10 mg/mL, Richter Pharma, Laboratorios Karizoo, Caldes de Montbui, Spain) combined with 4 mg detomidine hydrochloride i.v. (10 mg/mL, Domosedan, Orion Pharma, Barcelona, Spain) in the stall before the mare was taken to a set of stocks, regardless of weight. Once in the stocks, the rectum was emptied, and the perineum was cleansed using neutral soap and water. The vaginal vestibulum was scrubbed using cotton wool soaked in sterile saline. A 22 French-gauge Foley catheter was used to empty the bladder and left in situ during the whole OPU procedure. The mares were pre-medicated with 1.0 mg/kg flunixin-meglumine i.v. (50 mg/mL Finadyne, MSD Animal Health, Salamanca, Spain), 25 mg/kg procaine benzylpenicillin i.m. (300 mg/mL, Depocillin MSD Animal Health, Spain), and 6.6 mg/kg gentamycin sulphate (100 mg/mL, Gentavet, Fatro Ibérica, Barcelona, Spain). Just before OPU commenced, the mares received 0.1 mg/kg butylscopolamine bromide i.v. (Buscopan compositum: 4 mg/mL butylscopolamine and 500 mg/mL metamizole, Boehringer Ingelheim, Barcelona, Spain), which was repeated if needed during the procedure in case of rectal contractions. A second bolus of sedation was given (4 mg butorphanol and 2.5 mg detomidine) immediately before starting the procedure, and further boluses of the same dose were given if needed during OPU to maintain the plane of sedation.

**Figure 1:**
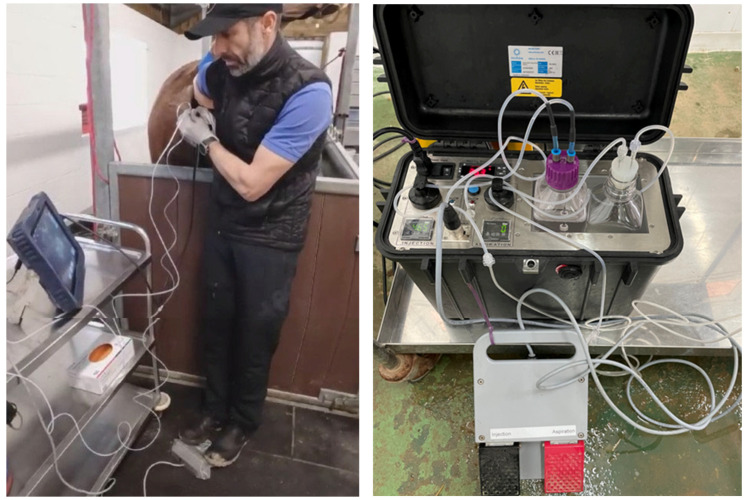
Example image of an OPU procedure being performed using a single-operator technique (left panel): The ovary (either side) is hold transrectally with the right hand and fixed close to the cranial vagina. The transvaginal OPU probe is held in the palm of the left hand, while the OPU needle is managed with the fingers of the left hand. The aspiration (right foot pedal) and injection (left foot pedal) of the medium into the follicles are controlled by the feet. The OPU pump (right panel) consists of the main pump with a temperature-controlled compartment for two 500 mL bottles: an injection (containing the flushing medium, pink lid) and a collection bottle (silicon cork lid), and a foot pedal with two different buttons (aspiration and injection). Vacuum and injection pressures are provided to each bottle by a tubing system originating from the aspiration and injection ports, which pass through a pinch valve controlled by each foot pedal, respectively.

In all experiments, the operator performing the procedure used a single-operator technique (Figure 1). The same operator held and fixed the ovary per rectum (right hand) and held a commercially available OPU probe (OPU probe and Exapad mini scanner, IMV technologies, L’Aigle, France) in the palm of the left hand while handling the needle in the thumb and index finger of the left hand at the same time, allowing for a needle rotation of approximately 90 degrees. A double lumen 12 G needle was used for all OPU procedures (Minitube equine OPU needle 12 G × 25″, Minitube Ibérica, Tarragona, Spain). It was connected to an OPU pump designed specifically for equine OPU (Figure 1) through a tubing system to allow aspiration and flushing of follicles and controlled by foot pedals (Figure 1) by the same operator (Minitube aspiration and flushing pump for equine OPU, 230 V, Minitube Ibérica, Tarragona, Spain). All antral follicles ≥3 mm (range from 3 to 45 mm in diameter) were punctured and aspirated and flushed 10 times using a commercial OPU medium containing heparin and PVA (Equiplus PVA 500 mL, Minitube Ibérica, Tarragona, Spain). The size of the punctured follicles was estimated using the scanner scale and registered by an assistant. Collection and media bottles of 500 mL were maintained at 35 C within the OPU pump’s temperature control compartment throughout the procedure.

### 2.3. Oocyte Search, Handling and Shipment

The collected fluid was poured through a sterile 70 mm embryo filter (Emcon; IMV Technologies Netherlands) immediately after the OPU procedure. The filtered contents were emptied into a sterile Petri dish, and oocytes were identified under a stereomicroscope (Zeiss Stemi 508; Zeiss, Madrid, Spain) by an experienced technician blinded to the experimental group, washed three times with modified HEPES-buffered synthetic oviductal fluid (mH SOF), transferred into a 2.5 mL cryovial containing mH SOF [15,16] and shipped overnight at 22 C in a polystyrene box designed for transporting organs for transplantation (ChillTherm; Sonoco Thermosafe, Mallow, Ireland) to a dedicated equine commercial ICSI laboratory for oocyte in vitro maturation (IVM), ICSI, and in vitro culture (IVC) of embryos and embryo cryopreservation.

### 2.4. IVM, ICSI and IVC of Shipped Oocytes

IVM of oocytes to the MII stage, Piezo-driven ICSI and IVC to produce blastocysts were performed as described previously [5]. ICSI was performed using frozen–thawed spermatozoa from stallions chosen by the mares’ owners, following gradient selection and swim-up [17]. Blastocysts were identified on Days 6, 7 or 8 after ICSI, cryopreserved by slow freezing in 10% glycerol and returned to the veterinary clinic in liquid nitrogen for storage and subsequent embryo transfer.

### 2.5. Experimental Design

**Figure 2:**
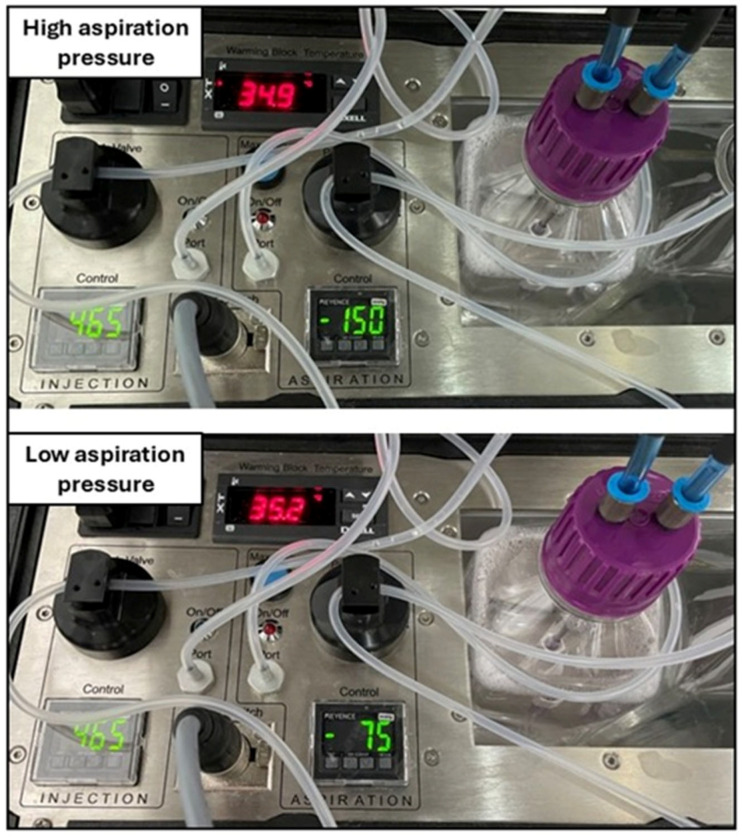
Details of the OPU pump with two different settings (aspiration control setting): high (upper panel) and low (lower panel) aspiration pressures.

The research design was a prospective study divided into three sequential experiments. Each experiment consisted of several OPU session days. In each OPU session, 4 to 8 mares were subjected to OPU in one day. In each experiment, two experimental groups were created according to one variable of the OPU technique: at the beginning of an OPU session, each mare was assigned to one experimental group in random order. The technician searching for the oocytes was blinded to the experimental group. All oocytes were handled and shipped to the ICSI laboratory in the same manner. The three sequential experiments are described as follows:

Experiment 1: The effect of aspiration pressure on oocyte recovery and in vitro embryo production was determined. Two aspiration pressure groups were created (Figure 2): high pressure (150 mmHg and 1.33 mL/s flow rate, *n* = 18 mares) and low pressure (75 mmHg and 0.75 mL/s flow rate, *n* = 18 mares). The flow rate was calculated by placing the OPU needle tip in a falcon tube filled with the flushing medium and measuring the time taken to aspirate 50 mL of the media. The pump was always placed at the same location (approximately 30 cm below the mare’s vulva). For each OPU session, the aspiration group (high or low) was alternated in sequential order for the following mares of the OPU session. The rest of the OPU parameters were kept constant (injection pressure of 465 mmHg, and needle rotation). The injection pressure used (465 mmHg) and the needle twisting during follicle flushing were similar in both groups.

**Figure 3:**
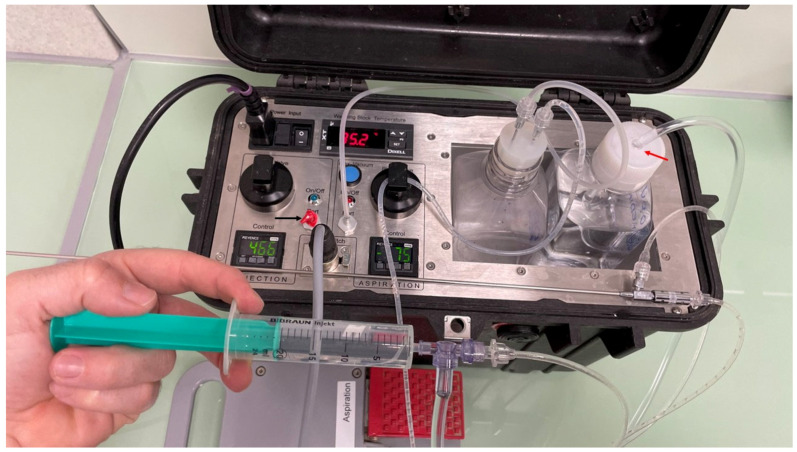
Example of the OPU pump with the injection port disabled (black arrow) by covering the exit with a plastic tape of red color (black arrow). The flushing of follicles is performed by injecting a media with a plastic syringe connected to a three-way automatic valve, which loads the flushing media from the bottle with an adapted lid (red arrow).

Experiment 2: The effect of the flushing method (manual vs. injection pump) on oocyte recovery was investigated. The same aspiration and injection pump (Minitube aspiration and flushing pump for equine OPU, 230 V, MInitube Ibérica, Tarragona, Spain) was used for both groups. However, in the manual injection group (*n* = 18), the injection port of the pump was disabled by covering the exit port with plastic tape (Figure 3); instead, a 20 mL rubber-free plastic syringe connected to a three-way automatic valve (controlled flushing set, Mila International, Florence, KY, USA) was used as the injection method to flush follicles. This method allowed refilling of flushing media and injection into the follicle via the syringe and tubing system, with a variable volume of injected medium according to follicle size (0.5 mL to 5 mL for follicles of 3 to >25 mm). The syringe was managed by a different operator. In the injection pump group (*n* = 18), the injection port was used as recommended by the manufacturer, using a constant injection rate (465 mmHg, injection flow rate of 1 mL/s) activated with a foot pedal. The injection pedal was activated until the follicle expanded to its original size. The aspiration pressure (75 mmHg flow rate of 0.75 mL/s) and the rest of the variables (needle twisting) were kept constant for both experimental groups.

Experiment 3: The effect of needle twisting on oocyte recovery was determined during OPU performed by a single-operator. Two experimental groups were created: the control group (*n* = 16), in which the needle remained still (no rotation) after follicle puncture (Supplementary Video S1), and the needle rotation group (*n* = 16), in which the needle was twisted approximately 90 degrees for 1–2 s (Supplementary Video S2) while the follicle collapsed. No attempt to massage the ovary or rotate the probe was performed in any group. As in the previous experiments, the allocation of each mare to the experimental group was randomized, and the technician searching for the oocytes was blinded to the experimental group. The rest of the OPU parameters were constant for both groups (aspiration pressure of 75 mmHg, flow rate of 0.75 mL/s, and injection pressure of 465 mmHg using the injection port with an injection flow rate of 1 mL/s).

### 2.6. Statistical Analyses

The following endpoints were registered and compared between the experimental groups in each experiment: mare age, number of aspirated follicles, percentage of aspirated follicles < 10 mm, oocyte recovery rate (number of oocytes divided by number of aspirated follicles), mean number of recovered oocytes, metaphase II rate ((MII rate): number of oocytes reaching MII (polar body) divided by the total of oocytes placed in IVM), cleavage rate (number of cleaved oocytes divided by the number of oocytes subjected to ICSI), blastocyst rate (number of blastocysts produced divided by the number of oocytes subjected to ICSI), number of embryos produces per mare and percentage of success (number of mares with at least one embryo produced divided by the total number of mares aspirated).

All data were computed using the statistical software Systat 13. Continuous data were presented as mean ± standard deviation (SD), while binary data were presented as percentages. Continuous data were tested for normality using the Shapiro–Wilk test. Normally distributed data were compared between experimental groups using the unpaired *t*-test, while not normally distributed data were compared using the Mann–Whitney non-parametric test. Binary data (success rate and oocyte per follicle) were tested using the chi-square test.

## 3. Results

The overall oocyte recovery rate of the study (all experiments, 104 mares) was 54.2%, with a mean number of aspirated follicles and recovered oocytes per OPU of 23.4 ± 7.8 (range of 8 to 45) and 12.7 ± 5.6 (range of 2 to 27), respectively, and a mean number of in vitro-produced embryos per OPU-ICSI session of 1.9 ± 1.6 (range of 0 to 7 embryos). Lastly, the OPU-ICSI success rate (mares with at least one embryo produced) was 78.8%.

### 3.1. Effect of Aspiration Pressure on Oocyte Recovery and Embryo Production (Experiment 1)

The detailed OPU and ICSI parameters of both experimental groups (high and low aspiration pressures) are shown in Table 1. The mean oocyte recovery rate in the high-pressure group (57.3 ± 16.7) was not different (*p* > 0.1) from the oocyte recovery rate of the low-pressure group (51.6 ± 18.9). However, oocytes aspirated with higher aspiration pressures (150 mmHg, flow rate of 1.33 mL/s) tended (*p* = 0.095) to have a lower MII rate (51.6 ± 12.1) than oocytes aspirated at a lower pressure (75 mmHg; 64.4 ± 18.5 MII rate) (Table 1). Furthermore, the blastocyst rate in the high-pressure group (20.6 ± 16.4) tended to be lower (*p* = 0.08) than the blastocyst rate in the lower-pressure group (31.4 ± 28.9; Table 1). The rest of the parameters were not affected by the aspiration pressure (*p* > 0.1; Table 1). Due to the tendency toward lower oocyte quality in the high-pressure group, subsequent experiments were performed using the low-pressure group (flow rate of 0.75 mL per second, 75 mmHg vacuum pressure).

### 3.2. Effect of Follicle Flushing Method on Oocyte Recovery (Experiment 2)

The injection port of the OPU pump used to flush follicles (controlled by a foot pedal) by the operator performing the OPU yielded an oocyte recovery rate (53.4 ± 14.0) similar (*p* > 0.1) to the oocyte recovery obtained from the manual method using a plastic syringe operated by an assistant (54.0 ± 18.9; Table 2). The rest of the OPU and ICSI parameters did not differ between the flushing methods (Table 2).

### 3.3. Effect of Needle Twisting During a Single-Operator OPU Technique on Oocyte Recovery (Experiment 3)

Surprisingly, the oocyte recovery in the control (no needle rotation) and needle rotation groups were similar (*p* > 0.1; Table 3), with an overall oocyte per follicle yield of 0.55 in both groups: 214/387 and 210/379 for the needle twisting and control groups, respectively. The twisting of the needle did not affect any of the ICSI parameters investigated (Table 3).

## 4. Discussion

This is the first study to investigate the effect of different OPU technique parameters on oocyte recovery and in vitro production of embryos in live mares aspirated using a single-operator OPU technique (where one person holds the ovary of the mare, transvaginal ultrasound probe, OPU needle and aspiration and injection pump).

One of the main hypotheses of the study was that aspiration pressure would increase oocyte recovery, as previously reported in bovine [12] and sheep [18] transvaginal follicle aspirations. However, this hypothesis was rejected, as the current study did not show any improvement in oocyte recovery in mares aspirated with a high flow rate (1.33 mL/s) and vacuum pressure of 150 mmHg. This observation agrees with a recent study on postmortem ovaries, in which a vacuum pressure of 300 mmHg and an aspiration flow rate of 1.9 mL/s did not increase the oocyte recovery rate (55.5%) compared with the recovery (58.4%) obtained with a much lower aspiration pressure (50 mmHg and 0.8 mL/s flow rate) [11]. It is possible that the stronger attachment of the equine oocyte to the follicle wall compared to the bovine follicle [10] accounts for a lack of difference in oocyte recovery between different vacuum pressures. Furthermore, a previous study on live mares [19] did not find any effect of aspiration pressure on oocyte recovery after aspirating preovulatory follicles 30 to 36 h after hCG administration: oocyte recoveries of 40%, 40% and 20% for aspiration pressures of 150, 280 and 400 mmHg, respectively. Although the reported study [19] and the current study are not comparable (immature vs. preovulatory follicles), aspiration pressure did not seem to influence oocyte recovery rate in both studies.

On the other hand, increasing aspiration pressure was associated with the recovery of more denuded oocytes (stripped from cumulus cells) in bovine [12,13,14,15] and porcine [20] OPU. Similarly, more denuded oocytes were obtained from aspiration of follicles of postmortem ovaries with 300 mmHg vacuum pressure (65.8% of denuded oocytes) than with aspiration pressure of 50 mmHg (41.7%) [11]. Unfortunately, in the current study, the oocyte morphology and evaluation of the number of cumulus cell layers surrounding the oocytes were not available. On the other hand, we observed a tendency for lower-quality oocytes from the high aspiration pressure group, as evidenced by lower maturation and blastocyst rate compared with the low aspiration pressure groups. However, this difference only approached significance (*p* < 0.1), and further research including a larger number of mares should be carried out to confirm this tendency observed in the current study. In bovine OPU-IVF, it is well accepted that oocytes recovered using high aspiration pressures result in fewer blastocysts due to certain damage during the aspiration process compared to procedures in which a low aspiration pressure is used to retrieve oocytes [13,21].

The second experiment in this study attempted to determine the effect of the flushing method to inject the medium into the follicle. The advantage of the manual method (plastic syringe) is that it is simple and inexpensive, but requires an extra operator. The pedal-operated pump eliminates the need for an extra operator and also provides a temperature-controlled space for placing the oocyte collection bottle. This is especially important when the OPU is performed during winter in a room that is not temperature controlled, as the oocytes should not be kept below 22 degrees Celsius to avoid oocyte damage and loss of developmental competence [22]. Nevertheless, a substantial initial investment is required to purchase a pedal-operated pump, some training is required to understand its features and operation, and specific silicone tubing and bottles are required to integrate it into the system. The results of the current study did not show any difference in oocyte recovery or other OPU parameters between both systems, the syringe system (manual injection of medium) and the pedal-controlled infusion pump. This is the first study to directly compare the two systems, which provide useful data for practitioners to choose from either system, according to their budget and staff availability.

With the manual syringe method, the exact volume that is injected into the follicle can be adjusted according to the estimated size of the follicle to be flushed. However, the injection pressure in each flush may vary, as it depends on the force used to push the syringe plunger with the hand. The injection pressure of follicle flushing was shown to affect the oocyte recovery rate in an equine postmortem OPU model: low and high injection pressures (200 and 800 mmHg) resulted in lower oocyte recovery rates (37 and 31%, respectively) than the recovery (47%) obtained with an intermediate injection pressure (465 mmHg) [11]. An elevated injection pressure (800 mmHg) was associated with an increased loss of oocytes outside the ovary and aspiration tubing [11,23].

No research has been done on the effect of injection volume according to follicle size on oocyte recovery. Using the pump method, the volume injected was controlled by adjusting the time during which the foot pedal was activated and by visualizing the re-expansion of the follicle to its original size. Therefore, it is unknown whether the volume injected in each follicle using both methods was comparable. However, since the recovery rate was similar in both groups, it seems that the injection pressure and volumes injected were equivalent with both flushing methods.

Lastly, Experiment 3 was designed to determine whether twisting the OPU needle during follicle aspiration to facilitate follicular wall scraping would increase oocyte dislodgment and recovery. Surprisingly, oocyte recovery was unaffected by needle rotation. This is in contrast with previous studies performed in slaughterhouse ovaries with a postmortem OPU model, in which needle twisting increased oocyte recovery by 15% [11] compared to the control group. A plausible explanation to account for the lack of difference in the current study is that in the reported study [11] with postmortem ovaries, the probe was held with a metal clamp while the operator had a free hand to hold and efficiently rotate the needle 180 degrees. On the contrary, in live mares with a single-operator OPU technique, as in the current study, the same operator needs to hold the probe and the needle with the same hand. This allows only partial rotation of the needle, using the thumb and index finger for 90 degrees at best, and as the fingers get tired (or numb), the intensity of needle twisting decreases. These differences in the intensity and degree of needle rotation between both studies could explain the lack of difference in oocyte recovery rate between the control and rotation groups in the current study.

Furthermore, previous studies in which a two-operator OPU technique was used [24,25,26,27,28] reported higher recovery rates compared to the current study. In those studies, one operator held the probe, while the second operator had a free hand to handle and rotate the needle more vigorously. On the other hand, a different approach to scraping follicles and aiding oocyte dislodgment during a single-operator OPU technique has been described. It consists of fixing the needle to the probe with the hand and rotating the whole probe instead of the needle and massaging the ovary at the same time [22] or rotating the needle and massaging the ovary at the same time [29,30,31]. In the current study, no attempt was made to massage or move the ovary during follicle scraping. However, it is unknown whether massaging the ovary during follicle scraping would increase the oocyte recovery rate using the single-operator technique described in this study. A previous study [29] using single-operator OPU technique with ovarian massage during needle rotation reported a higher oocyte recovery rate (116 oocytes from 166 follicles, 69.9% oocyte recovery rate) compared to that of the current study’s average of 54.2%.

In the current study, all oocytes obtained were submitted to ICSI. It is unknown how the OPU technique settings reported in the current study would influence the quality of oocytes if they were used for in vitro fertilization (IVF) instead. The IVF technique was not repeatable until recently, when an IVF protocol was described with reproducible results using fresh semen [3], and later using frozen semen [32], providing promising results to expand the possibilities of in vitro production of horse embryos.

## 5. Conclusions

In conclusion, vacuum pressure during follicle aspiration does not influence oocyte recovery (75 vs. 150 mmHg), but higher pressures tend to lower the maturation and blastocyst rate of oocytes. Therefore, there is no advantage of increasing the aspiration flow rate above 0.75 mL/s. The relationship between aspiration pressure and oocyte developmental competence should be confirmed with a larger number of mares and wider ranges of aspiration flow rates. Furthermore, when a single operator performs the OPU (holding the ovary and handling the needle simultaneously) without massaging the ovary, needle rotation to scrape the follicle wall does not improve oocyte recovery. This may indicate a poor scraping technique due to the difficulty in holding the probe and needle at the same time with one hand to rotate the needle efficiently.

## Figures and Tables

**Table 1:** Effect of aspiration pressure on OPU and ICSI parameters (Experiment 1).

Vacuum Pressure	Mares (*n*)	Donor Age (Years)	Flow Rate (mL/s)	Aspirated Follicles	Follicles < 10 mm (%)	Recovered Oocytes	Oocyte Recovery (%)	Overall Oocytes per Follicle	MII Rate (%)	Cleavage Rate (%)	Blastocyst Rate (%)	Embryos per Session	Success (%)
150 mmHg	18	12.1 ± 5.7 (4–24)	1.33	25.1 ± 7.6 (12–38)	70.4 ± 22.1 (10.5–100)	14.2 ± 5.7 (5–26)	57.3 ± 16.7 (25–66.7)	256/451 (0.57)	51.6 ± 12.1 ^a^ (25.3–70.4)	77.2 ± 15.7 (40–100)	20.6 ± 16.4 ^a^ (0–50)	1.61 ± 1.3 (0–4)	72.2
75 mmHg	18	12.7 ± 5.8 (5–25)	0.75	21.1 ± 8.2 (13–45)	66.8 ± 20.2 (37–100)	11.2 ± 6.1 (2–25)	51.6 ± 18.9 (14.3–88.2)	201/379 (0.53)	64.4 ± 18.5 ^b^ (33.3–100)	85.8 ± 16.8 (50–100)	31.4 ± 28.9 ^b^ (0–100)	1.89 ± 1.8 (0–7)	77.7

The high aspiration pressure group tended (*p* < 0.1; ^a,b^) to have a lower maturation rate (MII) and blastocyst rate than the low aspiration group. All mares’ follicles were aspirated using the same equipment, and every antral follicle aspirated was flushed 10 times using the injection port of the pump activated by a pedal (465 mmHg; injection flow rate of 1 mL/s), while the needle was twisted to aid follicle wall scraping. The pump was placed 30 cm below the needle and probe. Success: OPU-ICSI sessions in which at least one blastocyst was obtained.

**Table 2:** Effect of follicle flushing injection method on OPU and ICSI parameters (Experiment 2).

Injection System	Mares (*n*)	Donor Age (Years)	Aspirated Follicles	Follicles <10 mm (%)	Recovered Oocytes	Oocyte Recovery (%)	Overall Oocytes per Follicle	MII Rate (%)	Cleavage Rate (%)	Blastocyst Rate (%)	Embryos per Session	Success (%)
Pump pedal	18	13.4 ± 5.3 (3–26)	23.4 ± 7.7 (8–37)	60.9 ± 21.2 (12.1–95.5)	12.2 ± 4.4 (6–19)	53.4 ± 14.0 (31.6–77.8)	220/421 (0.52)	61.6 ± 12.4 (37.5–83.3)	85.0 ± 17.6 (50–100)	29.1 ± 26 (0–100)	2.05 ± 1.8 (0–7)	80.0
Syringe	18	14.0 ± 5.9 (5–25)	23.2 ± 6.7 (12–36)	69.7 ± 21.9 (41.2–100)	12.3 ± 4.2 (5–20)	54.0 ± 18.9 (25–86.7)	222/417 (0.53)	60.5 ± 16.5 (33.3–100)	79.6 ± 19.9 (43–100)	30.1 ± 22.2 (0–80)	2.05 ± 1.4 (0–4)	75.0

None of the parameters investigated were different (*p* > 0.1) between follicles flushed with a plastic syringe and those flushed using the injection port of the pump controlled by a foot pedal. All mares’ follicles were aspirated using the same equipment, and every antral follicle aspirated was flushed 10 times while the needle was twisted to aid follicle wall scraping. The pump was placed 30 cm below the needle and probe with an aspiration vacuum pressure of 75 mmHg (aspiration flow rate of 0.75 mL/s). Success: OPU-ICSI sessions in which at least one blastocyst was obtained.

**Table 3:** Effect of needle twisting during aspiration to aid follicle wall scraping on OPU and ICSI parameters (Experiment 3).

Needle Twisting	Mares (*n*)	Donor Age (Years)	Aspirated Follicles	Follicles <10 mm (%)	Recovered Oocytes	Oocyte Recovery (%)	Overall Oocytes per Follicle	MII Rate (%)	Cleavage Rate (%)	Blastocyst Rate (%)	Embryos per Session	Success (%)
Yes	16	14.4 ± 4.9 (3–23)	24.2 ± 7.6 (12–35)	69.4 ± 20.9 (35.3–100)	13.4 ± 6.4 (4–27)	54.4 ± 16.8 (22.2–76.5)	214/387 (0.55)	60.9 ± 13.2 (32.1–76.9)	82.4 ± 18.6 (47.5–100)	23.8 ± 21.3 (0–66.7)	1.94 ± 1.8 (0–5)	75.0
No	16	13.9 ± 5.1 (3–22)	23.7 ± 9.3 (13–42)	62.3 ± 19 (15.3–88.2)	13.1 ± 6.6 (3–25)	54.6 ± 20.5 (14.3–80.5)	210/379 (0.55)	68.1 ± 15.5 (41.2–100)	81.9 ± 19.4 (45–93.7)	25.2 ± 17.4 (0–50)	1.86 ± 1.2 (0–4)	81.2

Needle twisting: the needle was twisted for 1–2 s in a 90-degree rotation while the follicle collapsed. In the control group (no needle twisting), the needle remained still (stationary) during follicle flushing. No attempt to massage the ovary or rotate the probe was made in any group. None of the parameters investigated were different (*p* > 0.1) between needle twisting and no rotation. All mares’ follicles were aspirated using the same equipment, and every antral follicle aspirated was flushed 10 times using the injection port from the pump controlled by a foot pedal (465 mmHg and 1 mL/s injection flow rate). The pump was placed 30 cm below the needle and probe with an aspiration vacuum pressure of 75 mmHg (aspiration flow rate of 0.75 mL/s). Success: OPU-ICSI sessions in which at least one blastocyst was obtained.

## Data Availability

The raw data supporting the conclusions of this article will be made available by the authors on request.

## Supplementary Materials

The following supporting information can be downloaded at: https://www.mdpi.com/article/10.3390/ani15060832/s1, Video S1. Experiment 3, control. Video S2. Experiment 3, rotation.
